# Tuberculosis among the Jews

**Published:** 1902-01-25

**Authors:** 


### TUBERCULOSIS AMONG THE JEWS.

The ramifications of the tuberculosis question are unending, and there are but few social problems which do not come into touch with it at one point or another. In his Reports on the Eleventh Census of the United States, Dr. John S. Billings showed that so far as death-rates could be taken as an indication, tuberculosis was less common among the Hebrew than among the general population. Thus among the Jews the death-rate from consumption in 1,000 total deaths was for males 36 67 and for females 34·02; while among the general population the corresponding rate was for males 108·79 and for females 146·12. This was a very striking difference and unless it could be shown that Jewish physicians had peculiar notions as to nomenclature in the registration of the causes of death among their co-religionists, it seemed to indicate that the Jews were peculiarly exempt from a disease which one might fairly expect to be especially prevalent among them. In a recent article on this subject, Dr. Maurice Fishberg has pointed out that almost all the conditions of life which are generally recognised as predisposing to tuberculosis are highly developed among the Jews. The stature of the Jew is inferior to that of other Europeans, his chest girth is less. Most Jews are town dwellers, tailoring and similar sedentary occupations are common among them; they work in "sweat-shops"; their homes are crowded and often dirty, while a large number of them deal in second-hand clothing, and are thus peculiarly exposed to infection by the bacillus. Again, many of them are very poor, and, as a race, they have been ceaselessly persecuted as far back as history takes us. Everything would seem to be in favour of the bacillus, yet we are assured, by those who ought to know—not in America alone—that the Jews are less liable than those around them to tuberculosis. Many reasons have been suggested for this curious condition of affairs. Dr. Fishberg attributes the comparative immunity of the Jew to the care which is taken, according to the tenets of his religion, in the selection and slaughtering of the meat he eats. Others have put it down to the fact that Jews are mostly engaged in occupations that require no exposure to the weather; others, again, have asserted that it is a habit with the Jews to wipe dusty surfaces with a damp cloth, and hence that they do not run such risk of inhaling germs, while we can quite imagine that Dr. Archdall Reid would hold that the very persecution to which they have been so long exposed, the life in crowded and walled-in ghettos, the constant weeding out of all who were incapable of accommodating themselves to the unnatural conditions of town life, and the perpetual intermarriage of the survivors in this struggle for existence, have had the effect of producing a race more or less immune to those deteriorating influences which prove so fatal by way of tuberculosis to the country-bred people from among whom our town populations are so constantly recruited. Others again have held that the comparative freedom of the Jews from tuberculosis is due to their (also comparative) freedom from alcoholism, and in support of this we may refer to what was said at the recent Congress of Tuberculosis by Professor Brouardel, who maintained that alcoholism was "the most potent factor in the propagation of tuberculosis." The whole problem is one of extreme interest. We will not take it upon ourselves to say even that it is a fact that tuberculosis does spare the Jew, or to what extent there is a due foundation for the general belief on the subject. What is evident, however, is that if there is any truth in this belief—a belief be it said which is vouched for by many well-known and highly accredited names—the problem of the prevention of consumption extends far beyond those mere details as to germs and infection with which so many people are now occupying themselves. The present Royal Commission which is investigating the possibility of tuberculosis infection passing from animals to men is engaged on a most interesting task; but whatever conclusions it may arrive at they will not solve the problem.

